# Group Decision-Making in Multi-User Immersive Virtual Reality

**DOI:** 10.1089/cyber.2020.0065

**Published:** 2020-12-14

**Authors:** Ivan Moser, Sandra Chiquet, Sebastian K. Strahm, Fred W. Mast, Per Bergamin

**Affiliations:** ^1^ Institute for Research in Open, Distance and eLearning, Swiss Distance University of Applied Sciences, Brig, Switzerland.; ^2^ Department of Psychology, University of Bern, Bern, Switzerland.; ^3^ Faculty of Psychology, Swiss Distance University, Brig, Switzerland.

**Keywords:** group decision making, virtual reality, computer-mediated communication, collaboration, hidden profile

## Abstract

Head-mounted displays enable social interactions in immersive virtual environments. However, it is yet unclear whether the technology is also suitable for collaborative work between remote group members. Previous research comparing group performance in nonimmersive computer-mediated communication and face-to-face (FtF) interaction yielded inconsistent results. For this reason, we set out to compare multi-user immersive virtual reality (IVR), video conferencing (VC), and FtF interaction in a group decision task. Furthermore, we examined whether the conditions differed with respect to cognitive load and social presence. Using the hidden profile paradigm, we tested 174 participants in a fictional personnel selection case. Discussion quality in IVR did not differ from VC and FtF interaction. All conditions showed the typical bias for discussing information that was provided for all participants (i.e., shared information) compared with information that was only disclosed to individual participants (i.e., unshared information). Furthermore, we found that IVR groups showed the same probability of solving the task correctly. Social presence in IVR was reduced compared with FtF interaction; however, we found no differences in cognitive load. In sum, our results imply that IVR can effectuate efficient group behavior in a modern working environment that is characterized by a growing demand for remote collaboration.

## Introduction

Groups typically exhibit serious problems finding the optimal solution in decision tasks.^1–3^ The ubiquity of spatially distributed teams in modern business has raised the question of whether remote collaboration through communication technology further exacerbates or facilitates group performance.^4^ Meta-analyses revealed that computer-mediated communication (CMC) does not alleviate the typical issues of face-to-face (FtF) group decision-making.^4,5^ On the contrary, previous research comparing CMC with FtF communication mostly revealed similar or sometimes even more pronounced susceptibility for suboptimal decisions due to biased information sharing. However, recent technological advancements create new opportunities for remote collaboration.

In contrast to more established forms of CMC (e.g., text chat or video conferencing [VC]), virtual reality enables social interactions in three-dimensional spaces. Low immersive virtual reality can be displayed on desktop computers and its potential for remote collaboration has repeatedly been investigated.^6–8^ High immersive virtual reality (IVR) can be generated using head-mounted displays (HMDs).^9^ Promising findings show that simulations of social interactions using computer-simulated virtual agents in IVR are beneficial and training effects can transfer to actual in-person interactions.^10–13^ In the same vein, it is conceivable that IVR could be successfully used in communication settings between human-controlled avatars.^14^

Thus, we set out to compare group collaboration in IVR with FtF interaction. Furthermore, we were motivated to draw a comparison with VC. VC is ubiquitous in meetings of spatially distributed team members^15,16^ and different characteristics of CMC have shown to affect information sharing in groups.^4^ Since HMDs are becoming increasingly affordable and consumer-friendly, it is crucial to assess the suitability of IVR to complement or even enhance current standards of CMC (i.e., VC).

For this purpose, we administered the established hidden profile paradigm.^17^ In this paradigm, each group member receives both shared (i.e., disclosed to all participants) and unshared (i.e., unique to each participant) information about a decision case. The paradigm was developed to study information exchange in groups and to assess its effect on decision quality (i.e., finding the correct solution). Typically, groups that discuss more intensely and groups that introduce more unshared information are more likely to find the optimal solution in a hidden profile task (for comprehensive reviews, see Refs.^5,18,19^).

The hidden profile paradigm has also been studied in the context of CMC.^4,5,19^ In a meta-analysis, Lu et al. found that on average CMC and FtF interaction did not differ in terms of both discussion and decision quality.^5^ In other words, groups pooled the same amount of unshared information and they were equally likely to solve the task. However, the reported effects varied greatly, with some studies reporting advantages, while others found disadvantages of CMC over FtF groups. Including a broader range of collaborative tasks, Mesmer-Magnus et al. found that the virtuality of CMC influenced information exchange.^4^ Higher degrees of virtuality (i.e., more abstract forms of communication) lead to more pooling of unshared information, but less information exchange in general. In contrast, virtuality did not affect decision quality.

Based on the results from these meta-analyses, we argue that it is crucial to compare IVR, VC, and FtF interaction with respect to other variables that might influence group performance. First, it is still unclear what led to the inconsistent results in terms of decision quality.^5^ Second, it is not known why the increased exchange of unshared information in CMC^4^ does not result in better performance. It is conceivable that the unique cognitive and social implications of different forms of CMC influence how efficiently each group integrates the exchanged information during discussion. In fact, there is an increasing interest in understanding the cognitive and social implications of modern CMC.^20–24^

The cognitive load theory (CLT) provides a useful theoretical framework to explain working memory demands during the execution of complex tasks.^25^ According to the theory, cognitive load may arise due to the nature of the task material itself (intrinsic cognitive load), construction of new schemata during the task (germane cognitive load), and additional unnecessary load caused by the manner in which the material is presented (extraneous cognitive load).

CLT has also been debated in the context of collaborative tasks,^24,26,27^ including IVR. On the one hand, some researchers have raised concerns about cognitive load in IVR,^28–30^ for example, Makransky et al. recently reported higher cognitive load in IVR versus desktop VR,^28^ which is consistent with the notion that rich multisensory stimulation in IVR might induce cognitive overload.^31^ On the other hand, IVR displayed on HMDs implies certain characteristics that might help in reducing cognitive load. Bricken hypothesized that IVR is characterized by natural semantics, that is, a three-dimensional interface that is coupled to natural behavior.^32^ This is important since less cognitive load has recently been reported in stereoscopic compared with nonstereoscopic VC.^33^ Unlike standard (i.e., nonstereoscopic) VC, IVR inherently enables spatially directed interactivity with other avatars.

Spatial interactivity is not only relevant with respect to cognitive load but also has an effect on social presence, that is, the perception of other people in a mediated environment.^34–36^ Social presence depends on the ability of CMC to convey nonverbal cues. Compared with VC, IVR allows for spatially directed mutual gaze and gestures, which have shown to support social presence.^37,38^ However, IVR currently lacks the ability to convey facial cues about the emotional state of the other group members, which might affect the sensation of social presence.^34,39,40^

In summary, the present study set out to investigate for the first time whether IVR affects group collaboration in a hidden profile task. We expected to find evidence that IVR is a suitable technology for remote group collaboration. In other words, we expected similar information exchange and group decisions in IVR compared with FtF interaction. More specifically, we hypothesized similar amounts of shared and unshared information to be discussed (i.e., discussion quality; **H1**) and no differences in solution rates (i.e., decision quality; **H2**). Furthermore, we considered that the use of IVR might entail multiple benefits compared with VC. We expected that spatial interactivity in IVR leads to decreased extraneous cognitive load (**H3**) and increased social presence (**H4**).

## Methods

### Participants

When planning the experiment, we aimed at a sample size that was comparable with the reported numbers in a meta-analysis of hidden profiles (*M* = 150.36, *SD* = 73.38).^5^ In the end, we managed to recruit 189 participants from a secondary school, a vocational college, and a university. Participants were familiar with each other and signed up for the study in groups of three. Five groups had to be excluded due to recording failure or nonattendance. The final sample consisted of 174 participants (mean age = 18.42, *SD* = 1.82, 68 females) who were randomly assigned to solve the hidden profile using IVR, VC, or FtF interaction. Forty-nine percent of the participants reported having existing, however, limited, experience with IVR (typically in a museum, an exhibition, or with a friend, etc.). No participant mentioned regular use of IVR, that is, HMDs. Informed consent was collected from all participants before the experiment. No financial incentives were provided for study participation. The experiment was conducted in accordance with the Declaration of Helsinki and was approved by the local ethics committee.

### Material

As a group decision task, we used the hidden profile paradigm from the study by Schulz-Hardt et al.^41^ In this task, groups need to identify the best candidate in a personnel selection case. Groups of three participants are instructed to study information about four candidates, A, B, C, and D. The full information set characterized each candidate by 10 attributes, which were of positive or negative valence (for more detail on item selection, see Schulz-Hardt et al.^41^). Being characterized by the most positive attributes, candidate C was the most favorable candidate.^a^

Each group member only received a subset of the full information set. This individual information set contained shared information (provided to all group members) and unshared information (only disclosed to individual participants). Crucially, the individual information set was deliberately misleading by means of obscuring the higher number of favorable attributes of candidate C over candidates A, B, and D. Therefore, the correct decision could only be found if the group members pooled enough individual information. A schematic representation of the full and individual information set is provided in Supplementary Figure S1.

For all participants, we assessed demographic information and administered the social presence scale from the study by Poeschl and Doering,^35^ which was developed for IVR scenarios. To estimate cognitive load, we used the SOS Questionnaire from the study by Swaak and de Jong,^42^ adapted and extended by Eysink et al.^43^ Only items measuring extraneous cognitive load were analyzed since we were interested in possible differences of the task-irrelevant load induced by IVR.

**FIG. 1.**
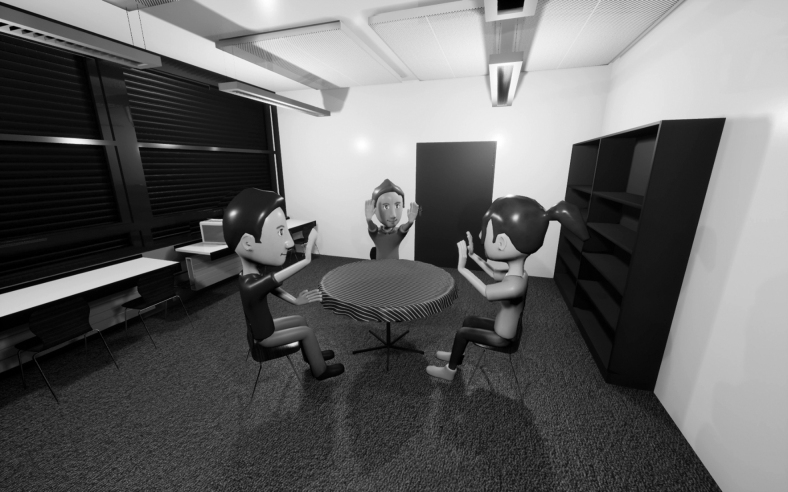
Three participants represented by virtual representatives (i.e., avatars) engaging in the group discussion.

FtF discussions took place in a laboratory equipped with a round table. They were recorded using a web camera at a resolution of 1,920 × 1,200 pixels, while screen-capturing software was utilized to record the discussions in IVR and VC. The virtual environment used in the IVR condition was modeled after the laboratory where the FtF discussions took place. The experiment was rendered on HTC Vive HMDs using the Unreal Engine 4.17.2. Visualizing an exemplary scenery, Figure 1 depicts the avatars within the virtual environment from a third-person perspective. We designed one female and one male avatar with differing hair color. The sex of the avatars always matched the participant. Hair color was randomly assigned (dark brown, blond, or red). All other features of the avatars were fixed (e.g., height, skin color, and hairstyle) and did not necessarily reflect the participant's true appearance.

The avatars were seated at a virtual table, which did not allow full-body movement. However, the avatars could move their head in sync with the HMD and their hands were tracked by hand controllers of the HTC Vive. Elbow position was computed by an inverse kinematics script of the Unreal Engine. Voice input to the microphone was managed by the Ultimate Voice Chat (Octagon Interactive Ltd.) plugin for the Unreal Engine. The auditory input was spatialized to mimic the spatial setting of the avatars (distributed equidistant around the table). Furthermore, signals from the microphone triggered a custom script to loop over a sequence of six different two-dimensional textures that represented the mouths of the avatars. The VC condition was performed using the same computer hardware and headphones as in the IVR condition. The group members were displayed on 19″ LCD computer screens using VC software (Adobe Connect Meeting).

### Procedure

The study was carried out in two appointments for each group. The first appointment served to familiarize participants with the VC software and the HMD for ∼10 minutes each. At the second appointment, participants were led into different rooms and sat down at the computer, where they read a cover letter introducing the decision case. Next, participants were prompted to memorize the candidate information sheet for 25 minutes since the provided sheets would not be available in the subsequent group discussion. Then, participants were asked to indicate their preferred candidate. Finally, the experimenter collected the information sheet and the discussion was prepared according to the randomly assigned condition. The groups started discussing using IVR, VC, or FtF interaction with no time restriction. After the discussion, the experimenter collected the group's decision and led each group member back to the separate rooms for the final questionnaires (social presence and cognitive load).

### Data analysis

For the analysis, we used the R package *brms* for Bayesian generalized (non)linear mixed models.^44^ Comprehensive details of the Bayesian analyses (i.e., sampling method and formal model notation of all statistical models, including prior distributions) are available in Supplementary Data.

Discussion quality was determined by the exchange of each candidate attribute (yes vs. no) during group discussion. Due to the dichotomy of the dependent variable, the odds of an attribute being introduced in the discussion were analyzed by means of a generalized linear mixed model (GLMM) with a binomial link function. Fixed effects included dummy-coded effects for IVR ($\beta_{IVR}$) and VC ($\beta_{VC}$) compared with FtF interaction. We also added $\beta_{Shared}$, a fixed effect for the difference between shared and unshared attributes. Furthermore, we included random intercepts to account for within-group variability. The first model (m1_discussed) only included additive effects for each predictor and was subsequently compared with a second model (m2_discussed), including interaction terms between the effects of condition and type of information ($\beta_{IVRxShared}$ and $\beta_{VCxShared}$).

Decision quality was defined as a dichotomous variable expressing the correctness of each group decision (correct vs. incorrect decision). It was predicted using a general linear model (GLM) with a binomial link function (m1_decision). We were primarily interested in differences between the three conditions. Thus, our model included two fixed effects $\beta_{IVR}$ and $\beta_{VC}$, which referred to the dummy-coded effects of IVR versus FtF interaction and VC versus FtF interaction, respectively. We were interested in whether the odds of providing a correct answer differed in IVR and VC groups compared with FtF groups (i.e., the reference category).

## Results

Below, we summarize the estimates (posterior means and 95% credible intervals [CrIs]) of the Bayesian analyses. The complete analysis (i.e., coefficient tables for all models) can be found in Supplementary Table S1.

### Discussion quality

**FIG. 2.**
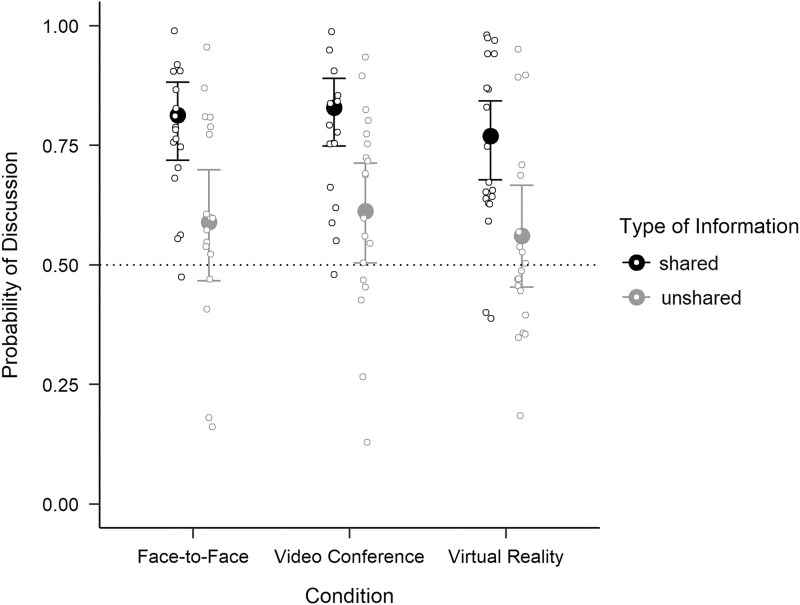
Posterior means and 95% credible intervals for the estimated probability for shared and unshared candidate attributes being discussed in face-to-face interaction, video conferencing, and immersive virtual reality. *Hollow circles* represent the raw values, that is, proportions of discussed candidate attributes for each group.

The first Bayesian GLMM (m1_discussed) modeled the introduction of candidate attributes during discussion as a function of condition (IVR vs. VC vs. FtF interaction) and type of information (shared vs. unshared). The posterior distributions of the GLMM revealed no difference in the proportion of candidate attributes that were exchanged in VC versus FtF interaction ($\beta_{VC}$ = 0.09; CrI = [−0.57 to 0.72]) and in IVR versus FtF interaction ($\beta_{IVR}$ = −0.18; CrI = [−0.78 to 0.46]). In contrast, the 95% CrI for the effect of type of information did not include zero ($\beta_{Shared}$ = 1.06; CrI = [0.86 to 1.26]). Shared candidate attributes (*M* = 0.81; CrI = [0.73 to 0.87]) were more likely to be introduced during group discussion than unshared attributes (*M* = 0.59; CrI = [0.48 to 0.69]). The second model (m2_discussed), including the interaction terms between condition and type of information, showed that the difference between the discussion of shared and unshared information was comparable between VC and FtF interaction ($\beta_{VCxShared}$ = 0.02; CrI = [−0.50 to 0.54]) and between IVR and FtF interaction ($\beta_{IVRxShared}$ = −0.14; CrI = [−0.65 to 0.36]). The posterior estimates, including 95% CrI for all combinations of condition and type of information, are visualized in Figure 2.

### Decision quality

The posterior estimates of the GLM (m1_decision) revealed no effect for VC versus FtF interaction ($\beta_{VC}$ = 0.39; CrI = [−1.19 to 1.95]) and IVR versus FtF interaction ($\beta_{IVR}$ = 0.98; CrI = [−0.47 to 2.53]). The probability of providing a correct answer did not differ between IVR (*M* = 0.40; CrI = [0.20 to 0.62]), VC (*M* = 0.27; CrI = [0.11 to 0.50]), and FtF groups (*M* = 0.20; CrI = [0.06 to 0.42]).

### Additional analyses

**FIG. 3.**
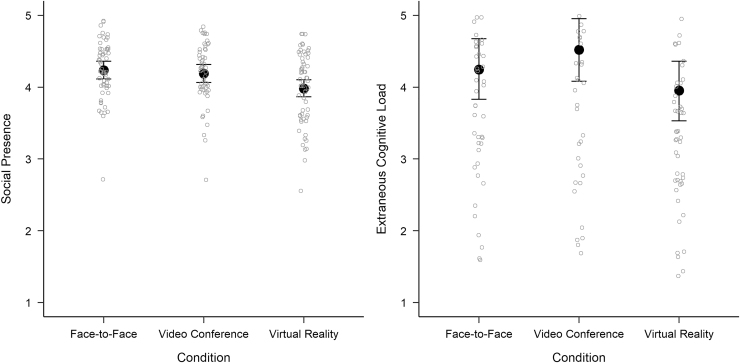
Posterior means and 95% credible intervals for the measures of social presence and extraneous cognitive load in face-to-face interaction, video conferencing, and immersive virtual reality. *Hollow circles* represent the raw values of all participants.

In addition to the analyses of decision and discussion quality, we were interested in whether the conditions differed with respect to social presence and extraneous cognitive load. Thus, both were entered as dependent variables in separate GLMMs (see Supplementary Data). The results showed that participants experienced lower social presence in IVR compared with FtF interaction ($\beta_{IVR}$ = −0.26; CrI = [−0.43 to −0.08]), whereas VC groups showed no difference from FtF groups ($\beta_{VC}$ = −0.05; CrI = [−0.23 to 0.13]). Changing the reference level to VC showed that IVR also leads to lower social presence than VC ($\beta_{IVR}$ = −0.21; CrI = [−0.38 to −0.04]). Regarding extraneous cognitive load, we found that IVR and VC did not differ from FtF interaction, ($\beta_{IVR}$ = −0.30; CrI = [−0.89 to 0.30]) and ($\beta_{VC}$ = 0.27; CrI = [−0.34 to 0.89]), respectively. Similarly, the 95% CrI of the effect of IVR versus VC did include zero ($\beta_{IVR}$ = −0.57; CrI = [−1.18 to 0.03]). Posterior estimates of the Bayesian analyses, including the raw data of social presence and extraneous cognitive load, can be found in Figure 3.

## Discussion

Using the hidden profile paradigm, we compared group decision-making in IVR with VC and FtF interaction. Consistent with earlier investigations of group collaboration in CMC^5,19^ and our hypotheses, we found no differences with respect to discussion quality (H1). Groups shared the same amount of information during discussion and exhibited the same degree of bias toward shared information. Additionally, the three conditions did not differ with respect to decision quality (H2). IVR showed comparable solution rates compared with VC and FtF interaction.

It is important to compare our results with previous findings regarding collaboration through CMC. It has repeatedly been found that the virtuality of CMC (i.e., abstractness of communication) influenced discussion quality.^4^ According to Kirkman and Mathieu,^45^ three dimensions determine the virtuality of CMC: (a) *use of virtual tools* (proportion of communication that takes place in the given medium), (b) *synchronicity* (absence of time lag in communication), and (c) *informational value* (transmission of information that is relevant for group performance). CMC tools that are similar with respect to these dimensions are expected to show comparable communication patterns compared with FtF interaction.^4^ Regarding our study, both the VC and IVR conditions were characterized by synchronous communication that took place exclusively in the mediated environment. Moreover, we argue that both technologies provided comparably rich multisensory input that mimicked FtF interaction. Participants in IVR were able to engage in spatially directed gaze and gestures, while they lacked the possibility to convey facial cues. Participants in VC could not engage in spatially directed behavior, but were able to convey and perceive facial cues in the live video stream. Therefore, the similar discussion quality across conditions is consistent with similar degrees of virtuality in VC and IVR compared with FtF interaction.

Although comparable in overall virtuality, it is still conceivable that the specific characteristics of each of the two technologies entailed certain advantages and drawbacks. For example, IVR and VC need to be discussed with respect to the cognitive load that they induce. There is an ongoing debate on whether the rich multisensory stimulation in IVR induces cognitive overload^28,30,31,46^ or whether it rather implies naturalistic interactions with the environment that are beneficial for information processing.^32,33,47–50^ Inconsistent with H3, we found no evidence for differences in extraneous cognitive load in IVR and VC. Thus, it is conceivable that the beneficial and detrimental aspects of IVR regarding cognitive load canceled each other out. Participants might have benefited from spatial interactivity while being confronted with unnecessary load due to rich stimulation in a highly immersive environment.

Spatial interactivity has also been related to social presence by means of higher social presence in spatial VC displays compared with standard two-dimensional VC.^37,38^ Our IVR condition allowed for spatially directed gaze and gestures, which were not possible in the VC condition. However, IVR lacked the ability to convey facial cues that carry information about the current emotional state of the other group members. This seemed to impair the sensation of social presence as we found lower levels in IVR compared with VC and FtF interaction. This finding was contrary to our expectations (H4) and highlights an important limitation of group collaboration in IVR. In fact, there is ample evidence that the transmission of facial expressions supports social presence.^51–53^ Nevertheless, it is noteworthy that no consistent links were found between social presence and performance in collaborative learning in experimental settings.^54,55^

To further assess the feasibility of IVR for remote collaboration, future studies should also include various aspects that relate to comfort and satisfaction of participants during the group interaction.^56^ Moreover, there is a general need for more investigations of long-term social interactions,^57,58^ not only in experimental but also in applied contexts. In fact, stronger and more evenly distributed connections between students in an undergraduate semester course were recently found when using a multi-user collaborative environment in desktop VR compared with an active control group.^59^ It is conceivable that the more immersive interaction with other avatars in IVR has a similar or even more pronounced effect on group cohesion. This is important since status inequality can negatively impact group decision-making.^19^ Unlike VC and FtF interaction, IVR offers various opportunities to manipulate social variables^60^ and was already used to counteract social biases.^61^

## Conclusions

Multi-user IVR can bridge the gap between the main advantages of IVR (simulation and manipulation of immersive three-dimensional objects) and the growing demand for effective collaboration of spatially distributed teams. Adding to the literature on immersive learning environments, we were able to demonstrate that IVR represents a feasible alternative to established methods of CMC. This creates new opportunities for remote work that relies on spatial interactivity within a virtual environment (e.g., engineering and training of health care professionals). IVR could complement existing forms of remote collaboration by enabling effective synchronous coworking in immersive virtual environments that were not achievable with existing forms of remote collaboration.

## Note

a. The correct solution (candidate C) was rotated in four versions of the information set. However, for the sake of simplicity, we always refer to the version that favored candidate C.

## Supplementary Material

Supplemental data
