# Supplementary material for: Group Decision-Making in Multi-User Immersive Virtual Reality
Source: Cyberpsychol Behav Soc Netw. 2020 Dec 14;23(12):846–53. doi: 10.1089/cyber.2020.0065 (PMC7757615; doi:10.1089/cyber.2020.0065)
Supplement: Supplemental data [file Supp_Data.pdf]

Supplementary Data

Data preparation, visualization, and analysis were conducted in R (3.5.1; R Core Team). The complete code is available on the Open Science Framework project page.

For the analysis, we took a Bayesian approach using the brms package for Bayesian generalized (non)linear mixed models.[S1] Markov Chain Monte Carlo sampling (MCMC) with 4 chains of 2,000 iterations was used to calculate posterior parameter estimates. Convergence of the chains was visually inspected using trace plots and assessed by means of the R-hat diagnostic. R-hat values larger than 1 were considered to reflect insufficient convergence of the MCMC chains.[S2]

Formal model notations of all statistical models are included below.

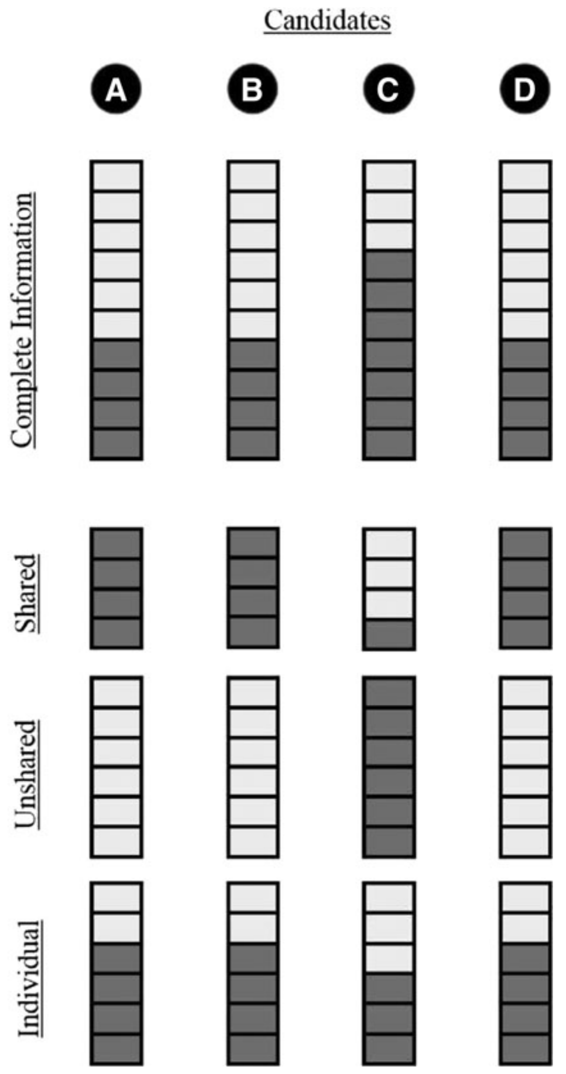

SUPPLEMENTARY FIG. S1. Schematic representation of the information set used in the hidden profile paradigm. *Dark gray squares* represent positive (i.e., favorable) and *light gray squares* represent negative (i.e., unfavorable) attributes about candidates A, B, C, and D.

Discussion Quality

m_1 discussed

$$\begin{array}{ll} \text{discussed}_i & \sim\text{Binomial}(n_i, p_i) \\ \text{logit}(p_i) & = \alpha + \alpha_{\text{group}[i]} + \beta_{\text{IVR}} \cdot \text{IVR}_i + \beta_{\text{VC}} \cdot \text{VC}_i \\ \alpha_{\text{group}} & \sim\text{Normal}\left(0, \sigma_{\text{group}}\right) \\ \alpha & \sim\text{Normal}(0, 10) \\ \beta_{\text{IVR}} & \sim\text{Normal}(0, 10) \\ \beta_{\text{VC}} & \sim\text{Normal}(0, 10) \\ \sigma_{\text{group}} & \sim\text{HalfCauchy}(0, 1) \end{array}$$

m2_discussed

$$\begin{array}{ll} \text{discussed}_i & \sim\text{Binomial}(n_i, p_i) \\ \text{logit}(p_i) & \begin{array}{l} = \alpha + \alpha_{\text{group}[i]} + (\beta_{\text{VC}} + \beta_{\text{VCxShared}} \cdot \text{Shared}_i) \cdot \text{VC}_i \\ + (\beta_{\text{IVR}} + \beta_{\text{IVRxShared}} \cdot \text{Shared}_i) \cdot \text{IVR}_i \end{array} \\ \alpha_{\text{group}} & \sim\text{Normal}(0, 10) \\ \alpha & \sim\text{Normal}(0, 10) \\ \beta_{\text{IVR}} & \sim\text{Normal}(0, 10) \\ \beta_{\text{VC}} & \sim\text{Normal}(0, 10) \\ \beta_{\text{IVRxShared}} & \sim\text{Normal}(0, 10) \\ \beta_{\text{IVRxShared}} & \sim\text{Normal}(0, 10) \\ \sigma_{\text{group}} & \sim\text{HalfCauchy}(0, 1) \end{array}$$

Decision Quality

m1_correct

$$\begin{array}{rr} \text{correct}_i & \sim\text{Binomial}(n_i, p_i) \\ \text{logit}(p_i) & = \alpha + \beta_{\text{VC}} \cdot \text{VC}_i + \beta_{\text{IVR}} \cdot \text{IVR}_i \\ \alpha & \sim\text{Normal}(0, 10) \\ \beta_{\text{VC}} & \sim\text{Normal}(0, 10) \\ \beta_{\text{IVR}} & \sim\text{Normal}(0, 10) \end{array}$$

Additional Analyses

Extraneous cognitive load

$$\begin{array}{rr} cl_i & \sim\text{Normal}(\mu_i, \sigma) \\ \mu_i & = \alpha + \alpha_{\text{group}[i]} + \beta_{\text{IVR}} \cdot \text{IVR}_i + \beta_{\text{VC}} \cdot \text{VC}_i \\ \alpha_{\text{group}} & \sim\text{Normal}\left(0, \sigma_{\text{group}}\right) \\ \beta_{\text{VC}} & \sim\text{Normal}(0, 10) \\ \beta_{\text{IVR}} & \sim\text{Normal}(0, 10) \\ \sigma_{\text{group}} & \sim\text{HalfCauchy}(0, 1) \end{array}.$$

Social presence

$$\begin{array}{rr} sp_i & \sim\text{Normal}(\mu_i, \sigma) \\ \mu_i & = \alpha + \alpha_{\text{group}[i]} + \beta_{\text{IVR}} \cdot \text{IVR}_i + \beta_{\text{VC}} \cdot \text{VC}_i \\ \alpha_{\text{group}} & \sim\text{Normal}\left(0, \sigma_{\text{group}}\right) \\ \beta_{\text{VC}} & \sim\text{Normal}(0, 10) \\ \beta_{\text{IVR}} & \sim\text{Normal}(0, 10) \\ \sigma_{\text{group}} & \sim\text{HalfCauchy}(0, 1) \end{array}.$$

Supplementary Table S1. Model Coefficients of All Statistical Analyses Included in the Article

Predictor	*Estimate*	*Est.Error*	*Q2.5*	*Q97.5*
Discussion quality				
m1_discussed				
Intercept	0.370	0.230	−0.080	0.820
VC	0.090	0.330	−0.570	0.720
IVR	−0.180	0.320	−0.780	0.460
Shared	1.060	0.100	0.860	1.260
m2_discussed				
Intercept	0.350	0.240	−0.120	0.830
VC	0.090	0.330	−0.560	0.730
IVR	−0.120	0.320	−0.750	0.510
Shared	1.110	0.190	0.730	1.480
VC:Shared	0.020	0.260	−0.500	0.540
IVR:Shared	−0.140	0.260	−0.650	0.360
Decision quality				
m1_correct				
Intercept	−1.410	0.590	−2.670	−0.310
VC	0.390	0.800	−1.190	1.950
IVR	0.980	0.760	−0.470	2.530
Additional analyses				
Social presence				
m_sp				
Intercept	4.240	0.060	4.120	4.360
VC	−0.050	0.090	−0.230	0.130
IVR	−0.260	0.090	−0.430	−0.080
Extraneous cognitive load				
m_cl				
Intercept	4.250	0.210	3.830	4.670
VC	0.270	0.310	−0.340	0.890
IVR	−0.300	0.300	−0.890	0.300

IVR, immersive virtual reality; VC, video conferencing.

Supplementary References

S1. Bürkner P. brms: An R package for Bayesian multilevel models using Stan. Journal of Statistical Software 2017; 80:1–28.

S2. Gelman A, Rubin DB. Inference from iterative simulation using multiple sequences. Statistical Science 1992; 7:457–511.
